# Survival of Resin-Bonded Fixed Metal-Ceramic Dental Prostheses Placed in the Anterior Region: A Descriptive Cross-sectional Study

**DOI:** 10.31729/jnma.6500

**Published:** 2021-05-31

**Authors:** Dinesh Sharma Bhusal, Sunita Khanal, Prakash Kumar Parajuly

**Affiliations:** 1 Department of Prosthodontics, Kantipur Dental College and Teaching Hospital, Basundhara, Kathmandu, Nepal; 2 Department of Community Dentistry, Kantipur Dental College and Teaching Hospital, Basundhara, Kathmandu, Nepal; 3 Department of Prosthodontics, College of Dental Surgery, BP Koirala Institute of Health Sciences, Dharan, Nepal

**Keywords:** *deep-bite*, *dental resisns*, *prosthesis survival*

## Abstract

**Introduction:**

Resin-bonded fixed dental prostheses have an advantage over conventional fixed dental prostheses in terms of conservation of tooth structure but many clinicians refrain from using this treatment plan over the concern of the retention of those prostheses. In anterior region, it has better survival rate because of the less masticatory force. This study was conducted to find out the survival of resin-bonded fixed metal-ceramic dental prostheses placed in anterior region of a tertiary care hospital.

**Methods:**

A descriptive cross-sectional study was carried out in a tertiary care hospital from September 2020 to February 2021. Ethical approval was taken from the Institutional Review Committee of Kantipur Dental College (reference number: 29/020). One hundred fifty-five patients who underwent prosthesis placement at least 5 years ago in the institution were identified from the hospital record and called, out of which only 80 agreed to be enrolled in the study. Consent was taken and data were collected using questionnaires filled by investigators and analyzed using the Statistical Package for the Social Sciences version 25. Point estimate at 90% Confidence Interval was calculated along with frequency and percentage for binary data.

**Results:**

Out of 80 patients, the prostheses survived in 72 (90%) (90% Confidence Interval 84.48- 95.51). The mean duration for which the prostheses survived in the mouth was 73.33±13.493 months with minimum 28 and maximum 98 months.

**Conclusions:**

This study showed that the survival after five years of resin-bonded fixed dental metal ceramic was high. The study's findings are comparable with international studies.

## INTRODUCTION

Removing healthy tooth structure (tooth preparation) for replacing a lost tooth is an aggressive treatment option.^1^ Presently available options for replacing a missing tooth/ teeth include the removable partial denture, partial and full coverage bridgework, resin-bonded bridgework and the single-tooth implant prosthesis.^2^ Since the introduction of resin-bonded fixed dental prostheses (RBFDPs) in the 1970s, this treatment option has become well accepted to replace missing anterior teeth.^3-5^

RBFDP is more conservative than conventional fixed dental prosthesis (FDP), but was thought to have less retention.^6-8^ However, its success when placed anteriorly is more than when placed posteriorly.^9^ Its longevity is affected by occlusion, number of pontics, position of the tooth in the arch, periodontal health, dentition, type of cement, operator's skill, and laboratory work.^10^ Despite being more conservative and suitable, it seems to be less utilized because of the fear of debonding.^11^

This study was performed to find out the survival rate of RBFPDs placed in the anterior region.

## METHODS

A descriptive cross-sectional study was carried out in the Kantipur Dental College and Teaching Hospital (KDCTH) from September 2020 to February 2021. Ethical approval was taken from the institutional review committee (IRC) of KDCTH (reference number: 29/020). One-hundred fifty five patients who had been treated at least 5 years ago with resin-bonded fixed partial denture from June 2012 to February 2016 were identified from the hospital record. All the patients were called by phone to participate in the study. Patients were called in the department and data were collected using questionnaires filled by the principal investigator. Patients who were given resin-bonded prosthesis other than metal-ceramic and RBFPDs placed in posterior region were excluded from the study. Convenience sampling was done and sample size was calculated as,


$$\begin{array}{ll} \text{n} & \text{= }{\text{Z}}^{\text{2}}\times \text{p}\times \text{q }/{\text{e}}^{\text{2}}\\ & \text{= }{\left(1.645\right)}^{\text{2}}\times {\left(0.5\right)}^{2}\times (1-0.5)/{(\text{0.10)}}^{\text{2}}\\ & \text{= }68 \end{array}$$

Where,

n = minimum required sample size
Z = 1.645 at 90% Confidence Interval (CI)
p = prevalence taken as 50% for maximum sample size
q = 1-p
e = margin of error, 10%

The calculated sample size was 68. Adding a 10% nonresponse rate, we arrived at a sample size of 75. Out of the 115 patients called, 80 participants who consented to follow-up were included in the study. An informed consent was obtained from all participants. The patients were examined primarily for the retention of the prosthesis, number of pontic teeth, type of overbite and number of retainers. This was done by filling the proforma by single investigator, the one who did the treatment for all those patients. The patients having more than 3mm of overbite were considered deep bite, <=3mm were considered normal bite and with no overbite were considered open bite.^12^ Retention was defined as a prosthesis that did not de-bond over the observation period, which also included any prosthesis that was extracted together with the abutment.^6^ Survival was defined as a prosthesis that was in situ at the time of review irrespective of its condition and cementation.^13^

The data was entered and analyzed in the Statistical Package for the Social Sciences version 25 using descriptive statistics. Point estimate at 90% Confidence Interval was calculated and results were expressed in terms of mean, standard deviation, frequency, and percentage wherever applicable.

## RESULTS

Out of 80 patients, metal framework survival after five years was seen in 72 (90%) (84.48-95.51 at 90% Confidence Interval) patients on recall examination (Table 1).

**Table 1:** Percentage of resin-bonded prostheses present during follow-up.

Variables	n (%)
Prosthesis Present	
Yes	72 (90)
No	8 (10)

The prostheses were in the mouth for the mean duration of 73.33±13.493 months with minimum 28 and maximum 98 months. Among the participants, 47 (58.75%) were males and 33 (41.25%) were females.

Of the total prostheses, 20 (25%) of the prosthesis had been re-bonded while 60 (75%) of the prosthesis never needed rebonding. Sixteen (20%) had re-bonding done once and 4 (5%) had twice (Table 2).

**Table 2:** Percentage of resin-bonded fixed dental prostheses needing re-bonding.

Variables	n (%)
Re-bonding Needed	
Yes	20 (25)
No	60 (75)
Number of Re-bonding	
Once	16 (20)
Twice	4 (5)

Out of 80 prostheses, 25 (31.25%) prostheses had been done in deep bite cases of which 8 (32%) of deep bite cases failed; 7 (28%) within the first five years. Fourty-six (57.5%) had been done in normal bite cases of which one (2.17%) failed. Nine (11.25%) prosthesis had been done in open bite cases of which one (11.11%) failed after five years (Table 3).

**Table 3:** Resin-bonded prostheses placed in different overbite conditions and their survival.

Prosthesis	Type of bite			Total n (%)
	Open n (%)	Normal n (%)	Deep n (%)	
≤Five Years	-	1 (1.25)	6 (7.50)	7 (8.75)
>Five Years	9 (11.25)	45 (56.25)	19 (23.75)	73 (91.25)
Total	9 (11.25)	46 (57.50)	25 (31.25)	80 (100)
**Survival**	**Among Open bite**	**Among Normal bite**	**Among Deep bite**	
	n (%)	n (%)	n (%)	
Yes	8 (88.89)	45 (97.83)	19 (76)	72 (90)
No	1 (11.11)	1 (2.17)	6 (24)	10 (10)

Of the total prostheses, 37 (46.25%) were placed in the upper arch. Three (3.75%) prostheses had one retainer, 61 (76.25%) had two, 10 (12.5%) had three, and four had six (7.5%) retainers. Of them, 51 (63.75%) had one pontic, 16 (20%) had two pontics, eight (10%) had three pontics, and five (6.25%) had four pontics (Table 4).

**Table 4:** Clinical characteristics of resin-bonded prostheses.

Variables	n (%)
Arch	
Upper	37 (46.25)
Lower	43 (53.75)
Number of Retainers	
One	3 (3.75)
Two	61 (76.25)
Three	10 (12.5)
Four	6 (7.5)
Number of Pontics	
One	51 (63.75)
Two	16 (20)
Three	8 (10)
Four	5 (6.25)

## DISCUSSION

As conservative treatment option to replace missing tooth/teeth, RBFPD is suitable for younger patients. It is a technique sensitive procedure because it requires proper planning of the clinical case and choice of materials. Abutment selection, retainer design, material used for retainer and pontics, the resin cement selection are some of the important factors to be considered. The occlusal scheme and force, the personality, physique, patient motivation, presence of parafunctional habits and daily oral hygiene of the patient should, of course, be considered in the prognosis. However, many of these factors are difficult to evaluate by studying the oral cavity, and it might be impossible to quantify them in retrospective studies.

The prostheses survived after five years in 90%. This result was consistent with the finding of the meta-analysis by Islam Abd Alraheam, et al.^11^ which included 38 studies published between 1965 to 2017. The study concluded that the five-year survival rate of 88.18% for the metal framework RBFPDs and 84.41% for nonmetal framework RBFPDs. The findings were also consistent with the meta-analysis by Thoma, et al.^9^ The meta-analysis of 29 studies reported on 2300 prostheses reported on survival rate on 2300 prostheses indicated an estimated survival rate of resin-bonded bridges of 91.4% (95% CI) after five years and 82.9 % (95% CI) after 10 years. He concluded despite the high rate of survival rate, the technical complications like de-bonding and minor chipping were frequent. Similar finding was observed by Michael George Botelho, et al.^6^ in a retrospective study evaluating the longterm clinical performance of resin-bonded fixed partial dentures. A total of 211 RBFPDs place in 153 patients were evaluated with success, retention and survival rate of 84.4%, 86.7% and 90% respectively with mean service life of 9.4 years.

An important finding observed in the study was that 32% of the RBFDPs done in deep bite cases failed in 8-year period; most of them within first five years. This clearly indicates the importance of proper case selection for RBFDPs.

This study has some limitations. It was carried out in a single hospital setting among a limited sample size. These findings may not be generalizable to all patients. Similarly, the study design did not allow us to find out the association between variables such as occlusion, number of pontics, position of the tooth in the arch, etc. with the longevity of the prostheses. Prospective and comparative studies with a follow-up time of 10 years or more is needed to fully assess the long-term outcomes of the resin-bonded fixed dental prostheses.

## CONCLUSIONS

Our study showed that the survival of resin-bonded fixed dental metal ceramic prostheses was high. Our findings are comparable with those from international studies. With proper case selection and operator's skill, resin-bonded fixed dental prostheses may be considered as valid minimally-invasive treatment alternatives to conventional fixed dental prostheses or single implant crowns. However, higher rate of failure in cases with deep bite clearly highlights the importance of proper case selection.
